# 1211. Incidence of All-Cause Community-Acquired Pneumonia in Ontario and British Columbia, Canada, 2002-2018; a Canadian Immunization Research Network (CIRN) study

**DOI:** 10.1093/ofid/ofab466.1403

**Published:** 2021-12-04

**Authors:** Sharifa Nasreen, John Wang, Jeffrey Kwong, Natasha S Crowcroft, Manish Sadarangani, Sarah Wilson, Allison McGeer, James D Kellner, Caroline Quach, Shaun Morris, Shelly Bolotin, Beate Sander, Monika C Naus, Linda Hoang, Frank Rudzicz, Shaza A Fadel, Fawziah Marra

**Affiliations:** 1 University of Toronto, Toronto, Ontario, Canada; 2 Public Health Ontario, Toronto, ON, Canada; 3 ICES, Toronto, Ontario, Canada; 4 University of British Columbia, Vancouver, British Columbia, Canada; 5 University of Calgary, Calgary, Alberta, Canada; 6 Montreal University, Montreal, Quebec, Canada; 7 Hospital for Sick Children, University of Toronto, Toronto, Ontario, Canada; 8 Public Health Ontario; University of Toronto, Toronto, Ontario, Canada; 9 University Health Network, Toronto, Ontario, Canada; 10 BC CENTRE FOR DISEASE CONTROL, Vancouver , BC, Canada; 11 British Columbia Center for Disease Control, Vancouver, BC, Canada

## Abstract

**Background:**

Community-acquired pneumonia (CAP) causes substantial morbidity and mortality. There is a lack of data on the comprehensive burden of CAP across the life span in Canada. We estimated the incidence of all-cause CAP in all age groups in Ontario and British Columbia (BC), Canada.

**Methods:**

We identified hospitalized and outpatient CAP episodes from the Discharge Abstract Database (DAD) and physician billing claims databases (Ontario Health Insurance Plan in Ontario and Medical Services Plan in BC) in both provinces. The National Ambulatory Care Reporting System was used to identify CAP episodes from emergency department visits in Ontario. CAP recorded with a primary or secondary diagnosis was identified using International Classification of Diseases 9 (480–486, 510, 513) and 10 (J10.0, J11.0, J12–J18, J86.9, J85.1) codes. We estimated the age and sex adjusted annual incidence of CAP overall, and by age groups (0–4, 5–17, 18–39, 40–64, 65–74, 75–84 and ≥85 years) according to routine childhood pneumococcal conjugate vaccine (PCV) immunization periods from 2005–2018 in Ontario and from 2002–2018 in BC. Poisson regression models were fitted with population denominators from Statistics Canada to estimate the incidence rates.

**Results:**

Ontario had 3,607,186 CAP episodes from 2005–2015 with a mean annual incidence of 2,801 (95% confidence interval [CI]: 2,748, 2,854) per 100,000 population; incidence declined from 3,077/100,000 in 2005 to 2,604/100,000 in 2010 before increasing to 2,843/100,000 in 2018. BC had 1,146,172 CAP episodes from 2002–2008, with a mean annual incidence of 2,146 (95% CI: 2105, 2189); the incidence increased from 2,005 /100,000 in 2002 to 2,199/100,000 in 2018. A high incidence of CAP was observed in children aged 0–4 years and older adults, particularly in adults aged ≥85 years in both provinces across all PCV program periods (Figure 1).

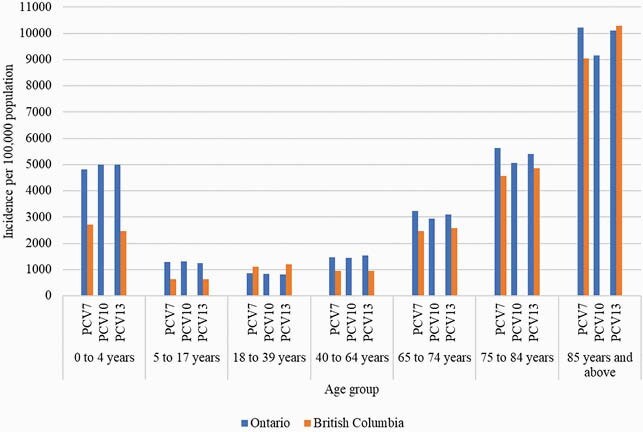

Figure 1: Age group-specific incidence of all-cause community-acquired pneumonia according to childhood pneumococcal conjugate vaccine (PCV) program periods in Ontario (PCV7 [1 Jan 2005–30 Sep 2009]), PCV10 [1 Oct 2009–31 Oct 2010] and PCV13 [1 Nov 2010–31 Dec 2018]) and British Columbia (PCV7 [1 Sep 2003–31 May 2010] and PCV13 [1 Jun 2010–31 Dec 2018]), Canada

**Conclusion:**

CAP continues to be a public health burden in Canada despite publicly funded pneumococcal vaccination programs. Ontario seems to have higher CAP burden than British Columbia that warrants further investigation. The youngest cohort of children and older adults contribute significantly to the CAP burden.

**Disclosures:**

**Manish Sadarangani, BM BCh, DPhil**, **GlaxoSmithKline** (Grant/Research Support)**Merck** (Grant/Research Support)**Pfizer** (Grant/Research Support)**Sanofi Pasteur** (Grant/Research Support)**Seqirus** (Grant/Research Support)**Symvivo** (Grant/Research Support)**VBI Vaccines** (Research Grant or Support) **Allison McGeer, MSc,MD,FRCPC,FSHEA**, **GlaxoSmithKline** (Advisor or Review Panel member)**Merck** (Advisor or Review Panel member, Research Grant or Support)**Pfizer** (Grant/Research Support, Scientific Research Study Investigator, Advisor or Review Panel member) **James D. Kellner, MD, FRCPC, FIDSA**, **Pfizer, Merck, GSK, Moderna** (Grant/Research Support) **Shaun Morris, MD, MPH, DTM&H, FRCPC, FAAP**, **GSK** (Speaker’s Bureau)**Pfizer** (Advisor or Review Panel member)**Pfizer** (Grant/Research Support) **Shaza A. Fadel, PhD MPH**, **Merck** (Other Financial or Material Support, Salary is paid by the University of Toronto via a donation by Merck to the Centre for Vaccine Preventable Diseases to support educational and operational activities.) **Fawziah Marra, BSc(Pharm), PharmD**, **Pfizer Canada** (Research Grant or Support)

